# Impact of Sample Storage Time and Temperature on the Stability of Respiratory Viruses and Enteric Viruses in Wastewater

**DOI:** 10.3390/microorganisms12122459

**Published:** 2024-11-29

**Authors:** Judy Y. Qiu, Richardson Mah, Logan A. Brand, Xiaoli Pang, Melodie Barnett, Mathew Diggle, Graham Tipples

**Affiliations:** 1 Public Health Laboratory, Alberta Precision Laboratories, Edmonton, AB T6G 2J2, Canada; richardson.mah@albertaprecisionlabs.ca (R.M.); mathew.diggle@albertaprecisionlabs.ca (M.D.); graham.tipples@albertaprecisionlabs.ca (G.T.); 2 Department of Laboratory Medicine and Pathology, University of Alberta, Edmonton, AB T6G 2R7, Canada; lbrand@ualberta.ca (L.A.B.); xlpang@ualberta.ca (X.P.)

**Keywords:** SARS-CoV-2, influenza virus, respiratory syncytial virus, enteric viruses, wastewater

## Abstract

Wastewater-based surveillance (WBS) has been widely used to track SARS-CoV-2 as well as many other viruses in communities during the COVID pandemic and post-pandemic. However, it is still not clear how temperature and storage time would influence the stability of viruses in wastewater. In this study, we assessed the stability of SARS-CoV-2, pepper mild mottle virus (PMMoV), influenza viruses A (IAV) and B (IBV), respiratory syncytial virus (RSV), and enteric viruses in raw wastewater stored at room temperature, 4 °C, and −20 °C for 3 and 6 days. SARS-CoV-2, PMMoV, IAV, and enteric viruses were found to be stable up to 6 days after storing at room temperature or 4 °C. SARS-CoV-2 and RSV were more susceptible to freeze–thaw cycles compared to PMMoV and enteric viruses, which were relatively stable for up to 6 days stored at −20 °C. Low detection of IBV in wastewater made it difficult to evaluate the impact. Based on our findings, we conclude that short-term storage or transportation of wastewater samples within 6 days at ambient temperature or 4 °C is acceptable for the majority of these viruses. Freezing samples at −20 °C for even short periods is not recommended for WBS of respiratory viruses. The data obtained from this study can provide guidance for quality assurance purposes from the operational aspects of wastewater surveillance.

## 1. Introduction

Wastewater-based surveillance (WBS) has been extensively used to track the circulation of SARS-CoV-2 in the general population during the COVID pandemic and post-pandemic. Many studies have shown the correlation between the concentration of SARS-CoV-2 in wastewater and clinical cases [1,2,3]. Therefore, this approach has been widely applied as a complementary tool to monitor the prevalence and predict the trends of SARS-CoV-2 infection in communities and to support the public health decision making [4]. In addition to SARS-CoV-2, many other infectious viruses have also been assessed by WBS, such as influenza viruses, enteric viruses, poliovirus, and monkey pox [5,6,7,8].

A key advantage of WBS on human infectious viruses over clinical testing is no selection bias, with comprehensive capture of all infected cases no matter the symptoms. This is based on the fact that infected individuals can shed viruses in fecal matter or other bodily fluids that are discharged into wastewater. Therefore, understanding the stability of viruses in wastewater is critical for the application of WBS for reliable assessment of virus prevalence. Wastewater is a complex matrix containing various biological and chemical agents which can affect virus stability. Although numerous studies have been conducted in the area of WBS, there is still a lack of clear conclusions regarding the impact of sample storage on virus stability in wastewater. Temperature and storage time are the two important factors contributing to virus degradation. However, studies on SARS-CoV-2 showed substantially inconsistent results for different storage times and temperatures. Some studies found that the SARS-CoV-2 gene level remained stable after wastewater sample storage at 4 °C for 7, 10, 19, and up to 84 days [9,10,11,12,13,14], while others showed significant reduction of SARS-CoV-2 copy number in wastewater with 4 °C storage for over 24 h or a month [3,15]. Similar inconsistent results of SARS-CoV-2 were also reported for wastewater samples stored at −20 °C [11,12,14,15,16].

The application of WBS on influenza A virus (IAV), influenza B virus (IBV), and respiratory syncytial virus (RSV), the most common respiratory viruses associated with endemic seasonal respiratory illness activity, were also reported [6,17,18]. To date, only two studies have assessed the stability of IAV in wastewater with different storage temperatures and times [19,20]. No studies have been conducted regarding RSV stability in wastewater.

Human enteric viruses, including norovirus (NoV), adenovirus (AdV), rotavirus (RoV), sapovirus (SaV), astrovirus (AsV), and enterovirus (EV), are the common causes of acute gastroenteritis. Although WBS has been used to monitor the circulation of these viruses [21,22,23], there has been limited work exploring the stability of enteric viruses in wastewater [19], especially for a panel of enteric viruses.

The goal of this study is to evaluate the stability of SARS-CoV-2, fecal indicator pepper mild mottle virus (PMMoV), IAV, IBV, RSV, and enteric viruses in wastewater with different storage times and temperatures. The results from this study will fill the knowledge gaps outlined above for better utility of WBS and data interpretation.

## 2. Materials and Methods

### 2.1. Wastewater Sample Collection and Storage

**Figure 1:**
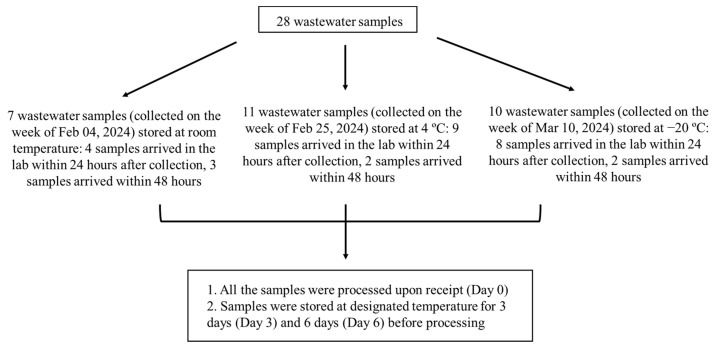
Flowchart of the wastewater sample storage and processing at different temperatures.

A total of 28 post-grit raw influent wastewater samples (500 mL of 24-h composite samples) were collected between February and March 2024 from 12 wastewater treatment plants (WWTPs) across Alberta, Canada. Samples tested at the same temperature condition were collected from the same week as indicated in Figure 1. Wastewater samples were shipped to the laboratory with an ice pack on the same day of collection. The majority of samples arrived in the laboratory within 24 h after collection, with a few samples arriving within 48 h. Upon receipt, wastewater samples were aliquoted into 100 mL per bottle and either processed immediately (Day 0) or stored at room temperature, 4 °C, or −20 °C for 3 (Day 3) or 6 days (Day 6) before processing for filtration. A flowchart of the wastewater sample storage and processing is outlined in Figure 1.

### 2.2. Virus Concentration and Viral Nucleic Acid Extraction

Virus concentration from wastewater was performed as previously described [16]. For samples stored at −20 °C, samples were thawed at room temperature on the day of processing. Briefly, 100 mL of wastewater sample was centrifuged at 4500× *g* for 10 min to remove solids. The supernatant was collected, transferred to the Centricon-plus 70 filter cup (30-kDa MWCO, Millipore, Burlington, MA, USA), and centrifuged at 3000× *g* for 10 min using a refrigerated centrifuge (Allegra X-15R, Beckman Coulter, Brea, CA, USA). The filtrate was discarded, and the same procedure was repeated until all of the 100 mL supernatant was filtered. After removing the filtrate collection cup and installing the concentration cup, the whole device was inverted and centrifuged at 800× *g* for 2 min. The concentrated sample was made up to a final volume of 1 mL with phosphate buffered saline (PBS) and processed for nucleic acid extraction.

Total nucleic acid was extracted from 400 μL of the concentrated sample using MagMAX-96 viral RNA isolation kit (Thermofisher, Waltham, MA, USA) on the automated KingFisher^TM^ Flex system (Thermofisher) and eluted at a final volume of 100 μL according to the manufacturer’s instructions.

### 2.3. One-Step Digital PCR (RT-dPCR) for SARS-CoV-2 and PMMoV

Multiplex dPCR assay was used to detect the *N1* and *N2* genes of SARS-CoV-2 and PMMoV on an Applied Biosystems QuantStudio Absolute Q digital PCR system (Thermofisher) according to the manufacture’s instructions. The dPCR reaction was performed in a total volume of 9 µL containing 4 × Absolute Q One-Step RT-dPCR Master Mix (Thermofisher), a 20 × Absolute Q dPCR SARS-CoV-2 wastewater surveillance kit, and 5 µL RNA template. The reaction mixture was pipetted into sample wells of the MAP16 plate (Thermofisher) followed by the addition of 15 µL of isolation buffer on top of each well. The dPCR settings were as follows: 10 min for reverse transcription at 55 °C, then 10 min at 96 °C for polymerase activation, followed by 40 cycles of 5 s at 96 °C for denaturation and 10 s at 60 °C for annealing and extension. The copy number of each target was analyzed and reported using the Absolute Q Analysis Software version 6.1.

### 2.4. Two-Step RT-qPCR for Enteric Viruses

Two-step RT-qPCR was performed for the detection of enteric viruses, including NoV GI and GII, AdV, RoV, AsV, SaV, and EV. Reverse transcription was carried out in a final reaction volume of 20 µL containing 5 mM DTT, a 20-unit RNaseOut^TM^ recombinant ribonuclease inhibitor, a 100-unit SuperScript^TM^ II reverse transcriptase (ThermoFisher), 2.5 mM each of dATP, dCTP, dGTP, and dTTP, 300 ng random primer, and 5 µL of nucleic acid extracts [24]. Nucleic acid was incubated at 95 °C for 5 min before adding the other reagents. The RT reaction was conducted for 1 h at 42 °C and 15 min at 70 °C. The qPCR reactions for the six targeted viruses were performed as previously described [25,26] and contained three duplex PCRs for NoV GI and GII, AdV and RoV, AsV and SaV, as well as a singleplex PCR for EV. The qPCR reaction was performed on an Applied Biosystems 7500 fast qPCR System (Applied Biosystems, Waltham, MA, USA) in a total volume of 10 µL containing 2 × TaqMan Fast Universal Master Mix (Thermofisher), 900 nM of each primer, 250 nM of a specific probe, and 3 µL of cDNA. Amplification consisted of initial incubation at 95 °C for 20 s followed by 45 cycles of 3 s at 95 °C and 30 s at 60 °C. A threshold of 0.05 was set for data analysis. A serial 10-fold dilution of a DNA fragment of NoV GII was used to generate the external standards to quantify the six viruses [26].

### 2.5. One-Step RT-qPCR for IAV, IBV, and RSV

One-step RT-qPCR was used to detect universal IAV, IBV, and RSV, including the commercial assay for IAV (Thermofisher), the CDC assay targeting the *NS1* gene of IBV [27], and a previous reported assay targeting the *L* gene of RSV A and B [28]. The qPCR reaction for IBV and RSV was performed in a total volume of 10 µL containing 4 × TaqMan Fast Virus One-Step RT-PCR Master Mix (Thermofisher), 800 nM of each primer, 200 nM of specific probe, and 5 µL RNA. The qPCR reaction for IAV was performed in a total volume of 20 µL containing 4 × TaqMan Fast Virus One-Step RT-PCR Master Mix, 20 × primer/probe mix (Thermofisher), and 5 µL of RNA. PCR amplification contained: 5 min for reverse transcription at 50 °C and 20 s at 95 °C for enzyme activation, followed by 45 cycles of 3 s at 95 °C and 30 s at 60 °C. A threshold of 0.05 was set for data analysis. The external standard curve was used to quantify the three viruses. All the qPCR amplification was performed on an Applied Biosystems 7500 fast qPCR System.

### 2.6. Statistical Analysis

Virus concentration was expressed as genome copies/100 mL wastewater after correction of the dilution steps and original volume of the wastewater sample, as previously described [16]. All virus concentrations were log_10_ transformed prior to data analyses. The copy numbers of Day 3 and 6 were compared to Day 0 using the paired Student *t*-test. The *p* value less than 0.05 was considered statistically significant.

## 3. Results

### 3.1. Stability of SARS-CoV-2 and PMMoV in Wastewater

**Figure 2:**
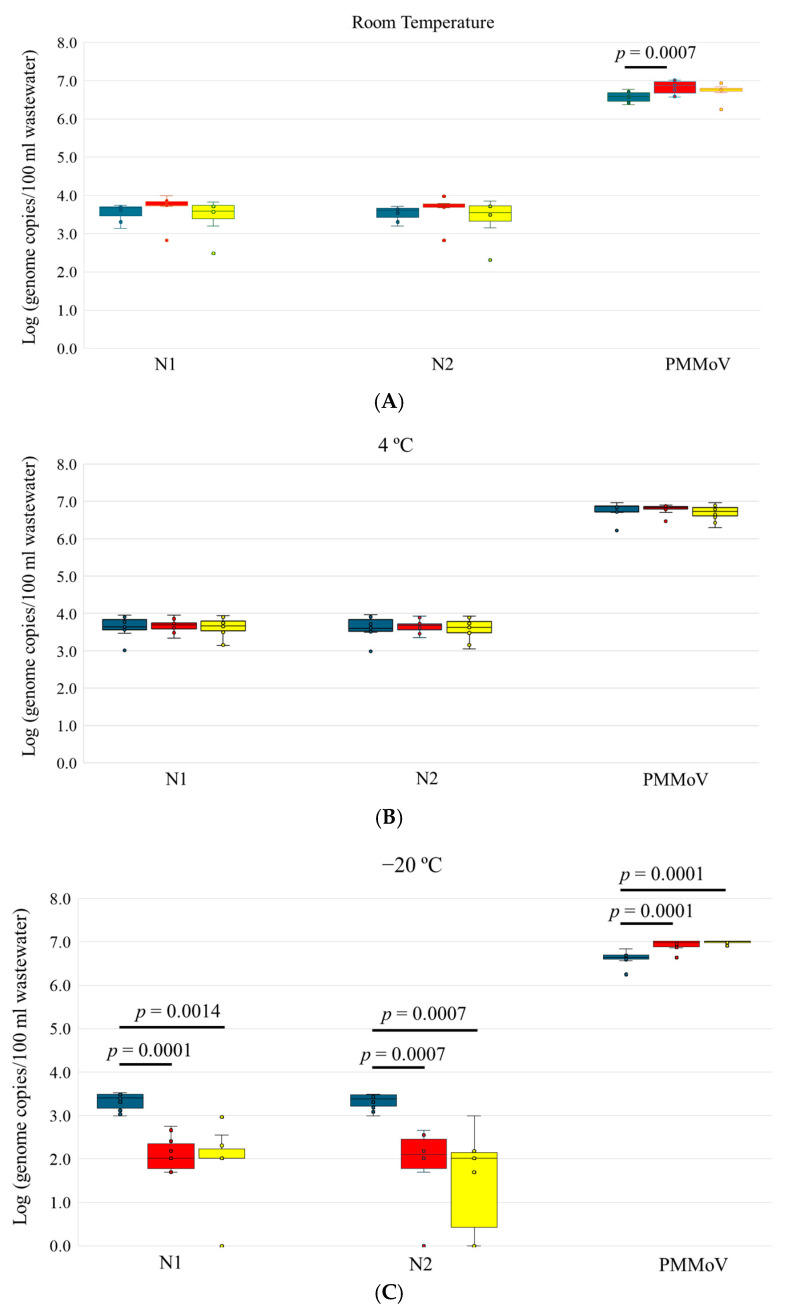
SARS-CoV-2 N1, N2, and PMMoV concentrations in wastewater samples at various storage times and temperatures. (**A**) Room temperature; (**B**) 4 °C; (**C**) −20 °C. Blue bars indicate the concentrations of the original samples processed upon arrival (Day 0); red bars indicate the concentrations after 3 days storage (Day 3); and the yellow bars indicate the concentrations after 6 days storage (Day 6). The *p* values are indicated for the groups with significant difference.

For wastewater samples stored at room temperature, SARS-CoV-2 N1 and N2 genes were detected in all 7 samples, and the concentration of both targets in wastewater did not vary significantly after 3 or 6 days of storage (Table 1, Figure 2A). Similar results for SARS-CoV-2 N1 and N2 were observed for wastewater samples stored at 4 °C for 3 and 6 days (Table 1, Figure 2B). However, for wastewater samples stored at −20 °C for 3 days, a 1–1.5 log reduction in N1 and N2 gene copy numbers was observed compared to Day 0 (*p* < 0.001), and N2 was not detected in 2 samples (Table 1, Figure 2C). For samples stored at −20 °C for 6 days, only 8 and 7 out of the 10 samples were detected with N1 and N2 genes, respectively. The copy numbers of N1 and N2 further reduced compared to Day 3, with 1.53 and 1.81 log reductions, respectively, compared to Day 0 (*p* < 0.01).

PMMoV was detected in all the samples with a high copy number under different storage times and temperatures (Table 1). Its gene copy number on Day 3 at room temperature (Figure 2A) and Days 3 and 6 at −20 °C was significantly higher than Day 0 at their respective temperatures (*p* < 0.001) (Figure 2C). There was no significant difference among other storage conditions for PMMoV.

### 3.2. Stability of IAV, IBV, and RSV in Wastewater

**Figure 3:**
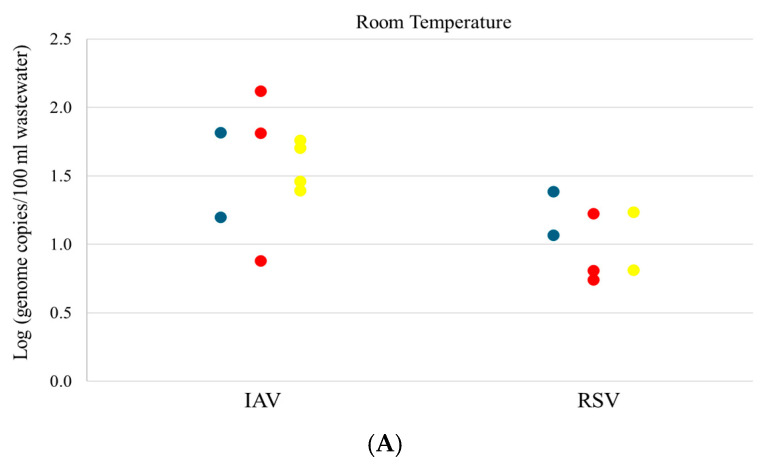

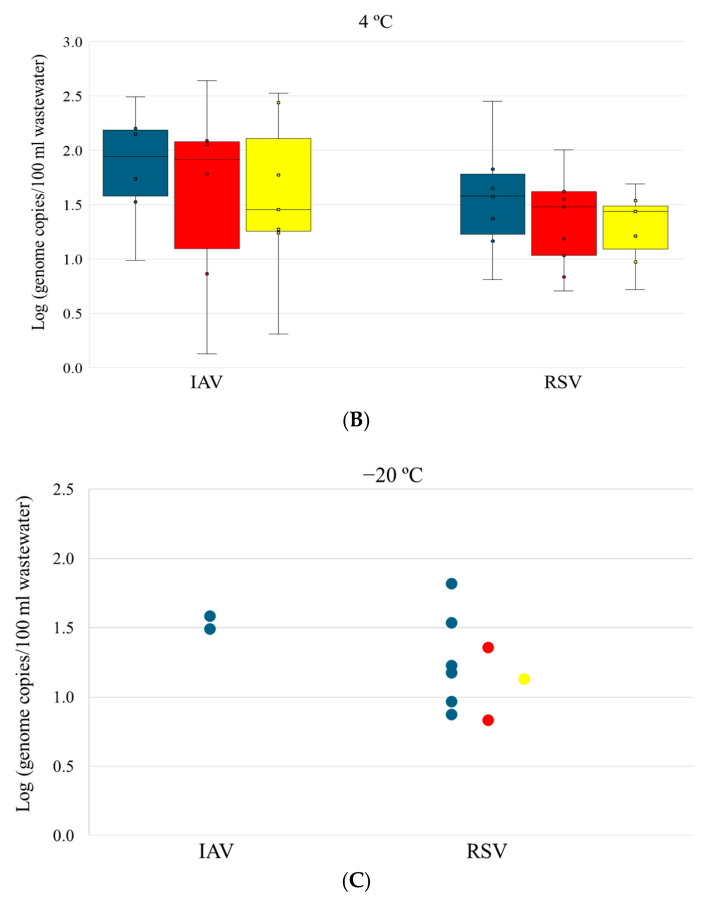
Influenza A virus (IAV) and RSV concentration in wastewater samples at various storage times and temperatures. (**A**) Room temperature; (**B**) 4 °C; (**C**) −20 °C. Blue dots/bars indicate the concentration in the original samples processed upon arrival (Day 0), red dots/bars indicate the concentration after 3 days storage (Day 3), and yellow dots/bars indicate the concentration after 6 days storage (Day 6).

The detection of IAV, IBV, and RSV was relatively low in wastewater compared to SARS-CoV-2 (Table 2). At room temperature storage, IAV was detected in only 2 samples on Day 0 and 3 and 4 samples on Days 3 and 6, respectively (Table 2, Figure 3A). At 4 °C storage, IAV was detected in 6 samples on Days 0 and 3 and 7 samples on Day 6 (Figure 3B). No significant variation was observed in the concentration of IAV after 3 or 6 days of storage compared to Day 0 at 4 °C or room temperature (Figure 3A,B). Overall, the copy number of IAV in wastewater was relatively low, with a mean concentration of less than 2 log genome copies/100 mL wastewater. At −20 °C storage, IAV was only detected in 2 samples on Day 0 while there was no detection on Day 3 or 6 (Figure 3C).

IBV was not detected in any samples at room temperature or −20 °C storage (Table 2). It was only detected in 2 samples on Day 0 and 1 sample each on Days 3 and 6 at 4 °C storage. Due to the low detection rate of IBV in this study, we are unable to evaluate the effect of sample storage time and temperature on IBV stability in wastewater.

RSV was detected in 2, 3, and 2 samples on Days 0, 3, and 6, respectively, at room temperature storage with relatively low concentration (Table 2, Figure 3A). At 4 °C storage, RSV was detected in 10, 9, and 7 samples on Days 0, 3, and 6, respectively (Figure 3B). No significant variation in the virus concentration was observed at room temperature or 4 °C. At −20 °C storage, RSV was detected in 6/10 samples on Day 0. However, this number reduced to 2 on Day 3 and 1 on Day 6 (Figure 3C). The overall concentration of RSV in wastewater was low, with less than 1.5 log genome copies/100 mL wastewater in most of the samples.

### 3.3. Stability of Enteric Viruses in Wastewater

**Figure 4:**
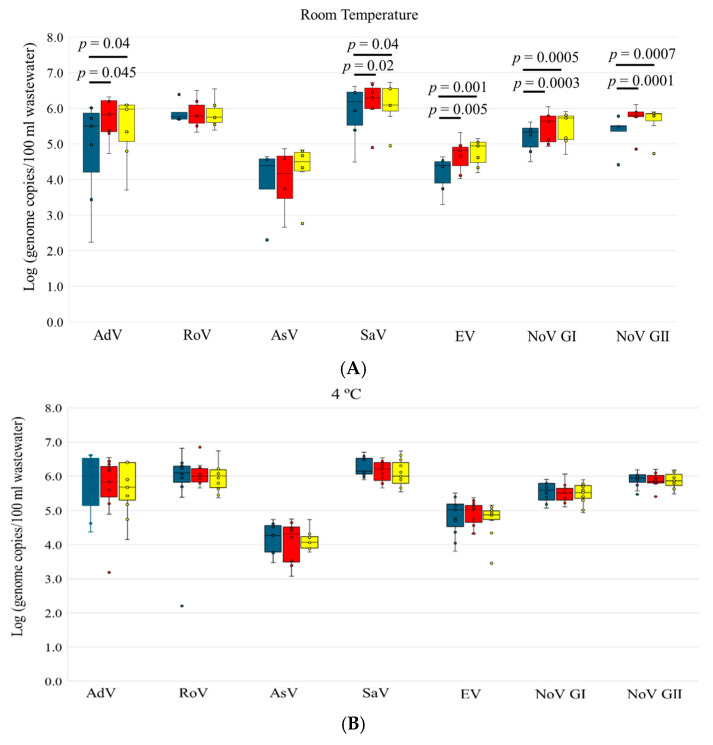

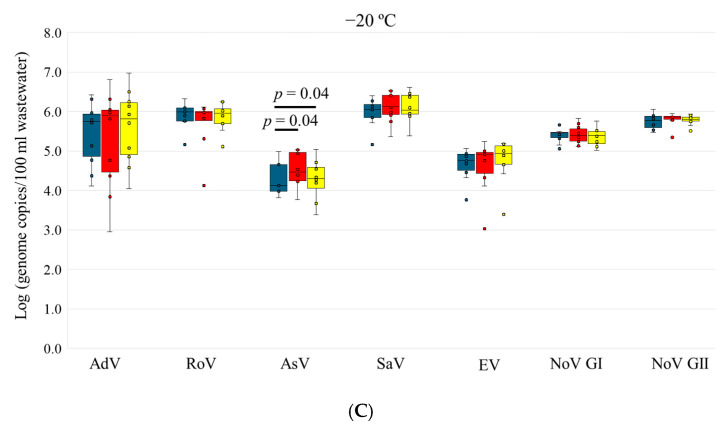
Concentrations of enteric viruses, including AdV, RoV, AsV, SaV, EV, NoV GI and GII, in wastewater samples at various storage times and temperatures. (**A**) Room temperature; (**B**) 4 °C; (**C**) −20 °C. Blue bars indicate the concentrations in the original samples processed upon arrival (Day 0), red bars indicate the concentration after 3 days storage (Day 3), and yellow bars indicate the concentration after 6 days storage (Day 6). The *p* value is indicated for the groups with significant difference.

Six enteric viruses were examined in this study (Table 3, Figure 4). AdV was detected in all the samples tested under different storage temperatures and times. The viral loads of AdV for samples stored at 4 °C and −20 °C on Days 3 and 6 were comparable to Day 0. Interestingly, AdV concentrations on Day 3 and 6 at room temperature were slightly but significantly higher than Day 0 (Figure 4A).

SaV, EV, and NoV GI and GII were detected in 27 out of 28 samples, with only one exception on Day 0 at room temperature. Similar to AdV, these three viruses were relatively stable after 3 or 6 days of storage at 4 °C and −20 °C without significant differences in viral load compared to Day 0 (Figure 4B,C). Another observation similar to that of AdV was that the concentrations of SaV, EV, and NoV GI and GII on Days 3 and 6 at room temperature were also significantly higher than Day 0 (Figure 4A).

The detection rate of RoV was slightly lower compared to AdV, SaV, EV, and NoV (Table 3), although the viral load of RoV was high in wastewater (~6 log genome copies/100 mL). No significant differences in the RoV concentration were observed between Day 3 and 6 and Day 0 for all three storage temperatures (Figure 4). AsV had the lowest detection rate among all the viruses tested, as well as the lowest concentration in wastewater (~4 log genome copies/100 mL). AsV concentration did not vary significantly for samples stored at room temperature or 4 °C for 3 or 6 days. However, AsV concentrations on Day 3 and 6 at −20 °C were slightly but significantly higher than Day 0 (Figure 4C).

## 4. Discussion

Viral decay remains a big challenge associated with WBS. In this study, we assessed the stability of different viruses in wastewater when the raw wastewater samples were stored at room temperature, 4 °C, and −20 °C for up to 6 days. This is the first comprehensive study involving major respiratory viruses, enteric viruses, and the fecal indicator PMMoV.

Overall, SARS-CoV-2 showed a very good detection rate at room temperature and 4 °C. Storing samples for up to 6 days under these two temperatures did not have a significant impact on the stability of SARS-CoV-2 in wastewater, suggesting that transportation of wastewater samples or sample storage before processing can be extended for up to 6 days at 4 °C or ambient temperatures without disrupting virus stability. This observation is in line with some previous findings that SARS-CoV-2 copy number remained stable after short-term storage of wastewater samples at 4 °C [9,13,14]. So far, limited data has been reported for wastewater samples stored at room temperature. Recent studies showed significant reduction in SARS-CoV-2 gene copy numbers after samples were stored at 20 °C for 2–10 days [29,30,31]. These contradictory observations may be due to the different processing procedures and the small sample sizes (1 or 2 samples) from those studies.

Our study also showed that freezing raw wastewater at −20 °C had adverse effects on the stability of SARS-CoV-2, suggesting that the freeze–thaw cycle can cause SARS-CoV-2 genome degradation; therefore, storage of wastewater samples at −20 °C should be avoided. This observation is supported by our previous study as well as those of others, all of which demonstrated the significant loss of SARS-CoV-2 signal in wastewater after freezing samples at −20 °C [11,13,14,16]. Based on these findings, preventing wastewater samples from freezing during transportation in extremely cold weather would be recommended to ensure the accuracy of tracking SARS-CoV-2 levels in wastewater.

Given that PMMoV is commonly used as a fecal indicator to normalize SARS-CoV-2 levels in wastewater [32,33], understanding the degradation of PMMoV is also crucial. In this study, the overall impact of storage time and temperature on PMMoV was minimal. This was also confirmed by other studies showing no significant decay signal of PMMoV RNA in wastewater at different temperature conditions (4 °C and 20 °C) [30,34]. The only significant effects were seen on Day 3 at room temperature and Days 3 and 6 at −20 °C, where PMMoV increased, in these cases, compared to Day 0. These variations may arise from the processing procedures, including concentration, extraction, and dPCR. Due to the high amount of PMMoV present in wastewater (up to 7 log copies/100 mL), dPCR detection may reach a plateau due to saturation of the signal, which may cause the variation.

For IAV, no significant impact on its stability when storing wastewater samples at room temperature or 4 °C for up to 6 days was observed in our study, indicating that IAV is relatively persistent in raw wastewater at 4 °C or ambient temperatures for short periods of time. So far, there has only been one study that showed no significant degradation of IAV after 14 days of storage at 4 °C or 25 °C [20], which was in agreement with our observation. For the samples stored at −20 °C, IAV was only detected in 2 samples on Day 0 with very low concentration but not on Days 3 or 6. Due to the low detection of IAV at −20 °C, it is hard to draw a conclusion in terms of the effect of −20 °C storage on IAV stability. No detection of IAV in samples stored for 3 or 6 days at −20 °C may be due to the variability of assay sensitivity for low viral load, which may also lead to the increased detection of IAV on Days 3 and 6 compared to Day 0 at room temperature. Further investigations under conditions of more pronounced influenza seasons with higher amounts of virus in wastewater and larger sample sizes would provide better insights on the impact of freezing wastewater samples at −20 °C on the stability of IAV.

IBV was only detected in the batch of 4 °C storage samples with very low frequency. This was consistent with previous findings in other countries that IBV was detected less compared to IAV [5,35]. However, Dumke et al. showed a higher detection rate (47%) of IBV than IAV (23%) in Germany [36]. These differences could be attributed to various factors such as sampling season, geographic locations, processing procedures, and wastewater matrix. To date, there has only been one study looking at the recovery of IBV from long-term storage of wastewater samples at −80 °C [19]. Similar to what we observed, IBV was only detected in one sample in their study and was not detected after 6 or 8 months of storage. With the limited detection of IBV in this study, the effect of storage time and temperature on the stability of IBV in wastewater is still not clear. Future studies on samples collected across different seasons, including the IBV peak season, would be useful to understand the persistence of IBV in wastewater.

This is the first study exploring the effect of storage time and temperature on RSV stability in wastewater. Our results showed that at room temperature, the detection rate of RSV remained stable after 3 or 6 days of storage. However, the average viral load was lower on Day 3 and 6 when compared to Day 0, even though the difference was not significant. A similar observation was made for samples stored at 4 °C. In addition, the detection rate decreased after 3 and 6 days of storage at 4 °C. These results provided limited information on the stability of RSV in wastewater after short-term storage at room temperature or 4 °C. Future studies with larger sample sizes would be necessary to further evaluate the stability of RSV in wastewater. Our study also showed that RSV detection reduced from 6/10 to 1/10 after 6 days of storage at −20 °C, indicating that freezing samples at −20 °C may cause degradation of RSV and impact its detection in wastewater.

In our study, enteric viruses remained relatively stable over time at all three temperatures. As non-enveloped viruses, enteric viruses are known to be more resistant to temperatures in environmental water compared to enveloped viruses such as SARS-CoV-2, influenza viruses, and RSV [19,37]. Of the enteric viruses, AdV was the most detectable virus with high abundance in wastewater, which is not surprising, as AdV is a DNA virus and is expected to be more persistent in environmental water compared to RNA viruses [38,39]. Interestingly, slightly higher concentrations of AdV, SaV, EV, and NoV GI and GII at room temperature, as well as AsV at −20 °C, were observed in Day 3 and 6 samples compared to Day 0. Similar findings were reported showing higher concentrations of NoV GI and GII after 6 months of storage of wastewater samples at −80 °C compared to the original samples [19]. Several factors may cause the variation, including the presence of inhibitory substances in the original samples, different viral decay rates, and various virus recovery rates from concentration procedures.

There are some limitations in our study. Due to the logistical constraints and staffing availability, we were only able to test limited number of samples for each storage condition during certain sampling period. Therefore, some viruses such as IAV, IBV and RSV might have low or undetectable level in wastewater, which hindered our investigation. More samples collected from different seasons would be needed to better understand the virus decay during storage and processing. In addition, we did not test sample storage for more than 6 days; thus, the impact of storing samples longer than 6 days on virus stability need further investigation.

## 5. Conclusions

This is the first comprehensive study evaluating the impact of sample storage time and temperature on the stability of respiratory viruses (SARS-CoV-2, IAV, IBV, RSV), PMMoV, and enteric viruses (NoV GI and GII, AdV, RoV, AsV, SaV, and EV) in wastewater. Our study showed that the enveloped viruses SARS-CoV-2 and RSV are more susceptible to freeze–thaw cycles compared to the non-enveloped viruses PMMoV and enteric viruses, which are relatively stable for up to 6 days of storage at −20 °C. While storing at room temperature or 4 °C, SARS-CoV-2, IAV, PMMoV, and enteric viruses were all stable for up to 6 days. Therefore, for virus monitoring in wastewater, short-term storage or transportation of wastewater samples at ambient temperatures or 4 °C is acceptable for the majority of these viruses. Freezing samples at −20 °C for even short periods is not recommended for WBS of respiratory viruses as this could cause virus degradation. The data obtained from this study can provide guidance for quality assurance purposes for the operational aspects of wastewater surveillance.

## Figures and Tables

**Table 1:** Comparison of the detection of SARS-CoV-2 N1/N2 gene and PMMoV in wastewater with different storage times and temperatures. Virus concentration is expressed as log genome copies/100 mL of wastewater. SD: standard deviation; R.T: room temperature.

		N1		N2		PMMoV	
		Detected Sample, *n*	Viral Load (Mean ± SD)	Detected Sample, *n*	Viral Load (Mean ± SD)	Detected Sample, *n*	Viral Load (Mean ± SD)
R.T storage	Day 0	7/7	3.56 ± 0.24	7/7	3.53 ± 0.2	7/7	6.57 ± 0.15
	Day 3	7/7	3.67 ± 0.39	7/7	3.64 ± 0.37	7/7	6.82 ± 0.19
	Day 6	7/7	3.45 ± 0.47	7/7	3.4 ± 0.53	7/7	6.71 ± 0.22
4 °C storage	Day 0	11/11	3.64 ± 0.27	11/11	3.63 ± 0.28	11/11	6.78 ± 0.20
	Day 3	11/11	3.66 ± 0.17	11/11	3.65 ± 0.17	11/11	6.80 ± 0.12
	Day 6	11/11	3.62 ± 0.27	11/11	3.60 ± 0.28	11/11	6.69 ± 0.20
−20 °C storage	Day 0	10/10	3.32 ± 0.20	10/10	3.32 ± 0.18	10/10	6.62 ± 0.16
	Day 3	10/10	2.22 ± 0.39	8/10	1.79 ± 0.99	10/10	6.93 ± 0.12
	Day 6	8/10	1.79 ± 0.99	7/10	1.51 ± 1.09	10/10	6.98 ± 0.04

**Table 2:** Comparison of the detection of influenza A virus (IAV), influenza B virus (IBV), and RSV in wastewater with different storage times and temperatures. Virus concentration was expressed as log genome copies/100 mL of wastewater. SD: standard deviation; R.T: room temperature. N/A: not available.

		IAV		IBV		RSV	
		Detected Sample, *n*	Viral Load (Mean ± SD)	Detected Sample, *n*	Viral Load (Mean ± SD)	Detected Sample, *n*	Viral Load (Mean ± SD)
R.T storage	Day 0	2/7	1.51 ± 0.44	0/7	N/A	2/7	1.23 ± 0.22
	Day 3	3/7	1.60 ± 0.65	0/7	N/A	3/7	0.93 ± 0.26
	Day 6	4/7	1.58 ± 0.18	0/7	N/A	2/7	1.02 ± 0.3
4 °C storage	Day 0	6/11	1.85 ± 0.54	2/11	2.22 ± 0.001	10/11	1.54 ± 0.45
	Day 3	6/11	1.59 ± 0.92	1/11	2.29	9/11	1.34 ± 0.43
	Day 6	7/11	1.56 ± 0.76	1/11	2.17	7/11	1.29 ± 0.34
−20 °C storage	Day 0	2/10	1.54 ± 0.70	0/10	N/A	6/10	1.27 ± 0.36
	Day 3	0/10	N/A	0/10	N/A	2/10	1.1 ± 0.37
	Day 6	0/10	N/A	0/10	N/A	1/10	1.13

**Table 3:** Comparison of the detection of enteric viruses, including adenovirus (AdV), rotavirus (RoV), astrovirus (AsV), sapovirus (SaV), enterovirus (EV), and norovirus (NoV) GI and GII in wastewater with different storage times and temperatures. Virus concentration is expressed as log genome copies/100 mL of wastewater. SD: standard deviation; R.T: room temperature.

		AdV		RoV		AsV		SaV		EV		NoV GI		NoV GII	
		Detected Sample, *n*	Viral Load (Mean ± SD)	Detected Sample, *n*	Viral Load (Mean ± SD)	Detected Sample, *n*	Viral Load (Mean ± SD)	Detected Sample, *n*	Viral Load (Mean ± SD)	Detected Sample, *n*	Viral Load (Mean ± SD)	Detected Sample, *n*	Viral Load (Mean ± SD)	Detected Sample, *n*	Viral Load (Mean ± SD)
R.T	Day 0	7/7	4.84 ± 1.46	5/7	5.88 ± 0.29	4/7	3.93 ± 1.1	6/7	5.89 ± 0.82	6/7	4.17 ± 0.53	6/7	5.17 ± 0.43	6/7	5.35 ± 0.48
	Day 3	7/7	5.71 ± 0.60	6/7	5.85 ± 0.43	4/7	3.96 ± 0.99	7/7	6.15 ± 0.62	7/7	4.68 ± 0.46	7/7	5.47 ± 0.45	7/7	5.72 ± 0.40
	Day 6	7/7	5.44 ± 0.92	6/7	5.84 ± 0.41	6/7	4.27 ± 0.78	7/7	6.11 ± 0.61	7/7	4.76 ± 0.38	7/7	5.45 ± 0.46	7/7	5.64 ± 0.42
4 °C	Day 0	11/11	5.78 ± 0.87	11/11	5.76 ± 1.24	7/11	4.16 ± 0.49	11/11	6.19 ± 0.36	11/11	4.83 ± 0.56	11/11	5.54 ± 0.29	11/11	5.90 ± 0.22
	Day 3	11/11	5.64 ± 0.97	10/11	6.07 ± 0.34	8/11	4.06 ± 0.64	11/11	6.15 ± 0.31	11/11	4.89 ± 0.37	11/11	5.50 ± 0.27	11/11	5.88 ± 0.21
	Day 6	11/11	5.65 ± 0.76	10/11	5.96 ± 0.42	8/11	4.12 ± 0.30	11/11	6.09 ± 0.40	11/11	4.63 ± 0.58	11/11	5.49 ± 0.31	11/11	5.87 ± 0.23
−20 °C	Day 0	10/10	5.44 ± 0.80	9/10	5.90 ± 0.33	5/10	4.31 ± 0.49	10/10	5.99 ± 0.36	10/10	4.66 ± 0.39	10/10	5.40 ± 0.18	10/10	5.75 ± 0.19
	Day 3	10/10	5.29 ± 1.24	9/10	5.69 ± 0.63	8/10	4.52 ± 0.45	10/10	6.11 ± 0.38	10/10	4.62 ± 0.65	10/10	5.41 ± 0.24	10/10	5.81 ± 0.17
	Day 6	10/10	5.60 ± 0.93	9/10	5.86 ± 0.37	8/10	4.27 ± 0.54	10/10	6.11 ± 0.37	10/10	4.76 ± 0.54	10/10	5.36 ± 0.23	10/10	5.78 ± 0.13

## Data Availability

The original contributions presented in this study are included in the article. Further inquiries can be directed to the corresponding author.
